# Two rare cases of gastric perforation caused by ingested metal bristle: the risk of barbecuing

**DOI:** 10.1093/jscr/rjaf877

**Published:** 2025-11-04

**Authors:** Amjad Mallisho, Theodora Dionysopoulou, Greta Kessler, Michael Drew Honaker, Andres Heigl, Anas Taha, Robert Rosenberg, Reinhard Stoll, Jasmin Zeindler

**Affiliations:** Faculty of Medicine, University of Basel, Basel, Switzerland; Department of Visceral Surgery, Cantonal Hospital Basel Land, Liestal, Switzerland; Department of Visceral Surgery, Cantonal Hospital Basel Land, Liestal, Switzerland; Department of Surgery, Brody School of Medicine at East Carolina University, Greenville, NC, United States; Department of Visceral Surgery, Cantonal Hospital Basel Land, Liestal, Switzerland; Faculty of Medicine, University of Basel, Basel, Switzerland; Department of Visceral Surgery, Cantonal Hospital Basel Land, Liestal, Switzerland; Department of Surgery, Brody School of Medicine at East Carolina University, Greenville, NC, United States; Department of Visceral Surgery, Cantonal Hospital Basel Land, Liestal, Switzerland; Department of Visceral Surgery, Cantonal Hospital Basel Land, Liestal, Switzerland; Faculty of Medicine, University of Basel, Basel, Switzerland; Department of Visceral Surgery, Cantonal Hospital Basel Land, Liestal, Switzerland

**Keywords:** gastric perforation, foreign body ingestion, laparoscopy, grill wire, metal bristle, CT imaging

## Abstract

Gastric perforation due to ingested foreign bodies is rare and often diagnostically challenging. We present two cases of patients presenting with acute abdominal pain after barbecuing in 2024. Both showed gastric perforation due to metal bristle ingestion from grill brushes attached to the food. Consecutively, diagnostic laparoscopy and recovery of the foreign body and gastric perforation repair with sutures were performed. These cases underscore the importance of imaging for operative planning, diagnostic persistence, and awareness of concealed gastric perforation due to foreign body ingestion. Utensils used for food preparation should be considered in differential diagnosis for bowel perforation and liver abscesses. Thin metal wires can detach, be ingested, and cause gastrointestinal complications.

## Introduction

Foreign body ingestion is a common clinical issue, yet gastrointestinal perforation remains rare, occurring in fewer than 1% of cases [1]. Most ingested foreign objects, including fish bones, toothpicks, and bristles, pass spontaneously without complication [2]. When perforation does occur, it most often involves anatomically narrow regions such as the ileocecal junction or rectosigmoid colon [3]. Gastric perforation is unusual, and posterior wall penetration into adjacent structures is exceptionally rare [4]. Few cases of metal bristle ingestion have been reported [5–7]. A recent commentary has even proposed banning wire-bristle grill brushes in light of such injuries [8].

Some patients may not recall the ingestion, especially with grill-cleaning bristles, making diagnosis even more difficult [9]. Here, we present two cases of gastric wall perforation by a metallic bristle and consecutive migration of these wires, successfully managed via laparoscopic retrieval.

## Case reports

### Case 1

A 44-year-old female presented to the emergency department with acute-onset, cramping abdominal pain for 24 hours after barbecuing. Symptoms were localized to the left upper quadrant without nausea, vomiting, or fever. On examination, localized tenderness with mild guarding was noted in the epigastric region.

**Figure 1:**
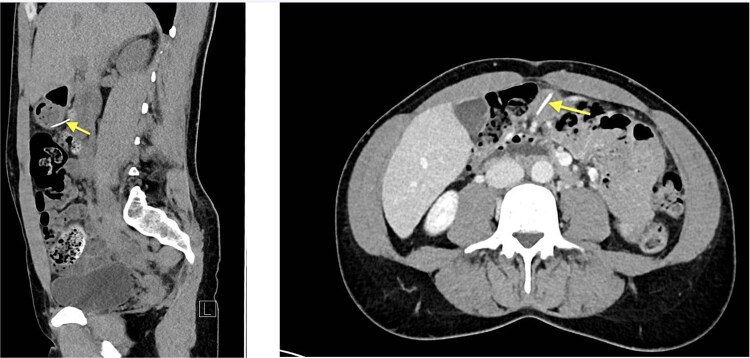
Abdominal CT (03.05.2024), hyperdense foreign body (metallic wire).

Initial laboratory parameters were unremarkable. Abdominal computed tomography (CT) (03.05.2024) revealed a linear, hyperdense foreign body (Fig. 1)—consistent with a metallic wire—penetrating the anterior gastric wall and abutting the dorsal rectus sheath left paramedian [10]. Minimal free fluid was noted, but no pneumoperitoneum. Two small hepatic lesions were incidentally noted, suggestive of hemangiomas.

Emergent gastroscopy showed no intraluminal foreign body. A single mucosal lesion on the posterior wall was thought to be iatrogenic. Diagnostic laparoscopy the same day was inconclusive, revealing only a superficial lesion at the distal anterior wall; no foreign object was retrieved. The lesion was sutured, and consecutive air testing didn’t show any leakage.

Repeat CT (05.05.2024) demonstrated the wire had migrated, now traversing the posterior gastric wall into the mesentery near the hepatic flexure. New gas collections in the Morrison pouch and right subdiaphragmatic space were noted [11].

**Figure 2:**
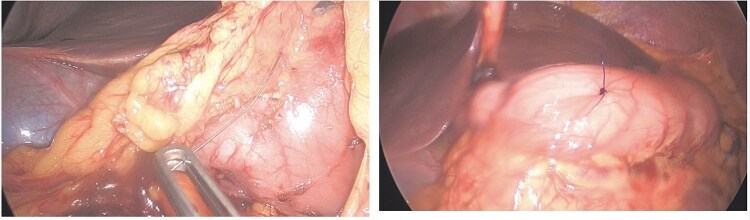
Case 1 intraoperative.

Given persistent pain and fever, a second-look laparoscopy was undertaken. Access to the lesser sac via the gastrocolic ligament revealed the metallic wire (Fig. 2), embedded near the pyloric region. It was successfully extracted without mucosal rupture or abscess formation.

The patient recovered without complications and was discharged on postoperative day four. Follow-up abdominal ultrasound confirmed the hepatic lesions as hemangiomas. No recurrent symptoms were reported at 1-month follow-up.

### Case 2

**Figure 3:**
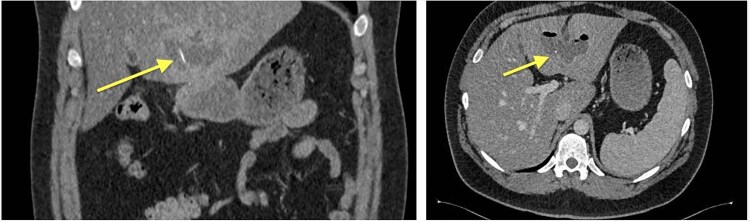
Abdominal CT, hyperdense foreign body (metallic wire).

Four months later, a 33-year-old man presented with fever and weakness of unknown origin for 5 days. He was barbecuing a lot in the last weeks, as he was on vacation. Epigastric tenderness was present at admission. Laboratory parameters showed elevated inflammatory markers. CT scan (Fig. 3) showed a liver abscess with a metal foreign body.

**Figure 4:**
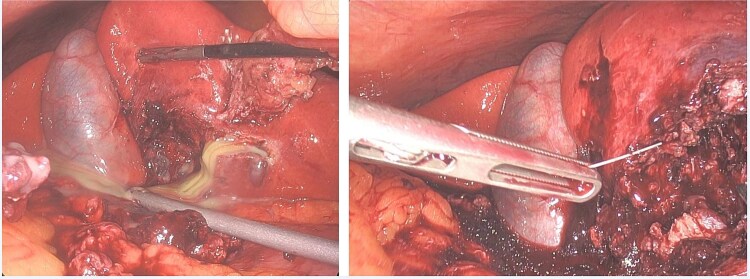
Case 2 intraoperative.

Eventually, diagnostic laparoscopy showed a small posterior gastric perforation next to the liver abscess with migration of the metal bristle into the liver, causing the abscess. Consecutively, surgical drainage of liver abscess, recovery of the foreign body, and gastric perforation repair with sutures were performed (Fig. 4). No postoperative complications occurred. The patient received intravenous antibiotic treatment for 10 days, followed by oral antibiotics for another 4 days. Follow-up showed decreasing CRP and WBC and rapid recovery after surgery. On the 4th postoperative day, the patient could be discharged.

## Discussion

Gastrointestinal perforation from ingested foreign bodies remains an uncommon but potentially serious complication, seen in <1% of ingestion cases [1]. While sharp objects such as toothpicks, fish bones, and grill wire bristles are more likely to cause perforation, their diagnosis is frequently delayed due to the absence of a clear ingestion history [2].

The stomach is an unusual site for perforation due to its thick muscular wall and spacious lumen. However, posterior wall perforations are particularly difficult to detect as they may lead to retroperitoneal, lesser sac involvement or liver abscesses without generalized peritonitis and unspecific symptoms [3].

CT imaging played a critical role in these cases, both in initial detection and in identifying positional change, which raised suspicion for perforation and mesenteric migration [4]. As emphasized in ESGE guidelines, persistent symptoms despite negative endoscopy or laparoscopy should prompt further imaging and potential reintervention [9].

Intraoperatively, direct access to the lesser sac allowed visual confirmation and safe extraction [10]. Literature supports laparoscopic over open retrieval when technically feasible, offering better visualization and reduced morbidity [11].

A growing number of cases implicate grill-cleaning wire bristles as culprits in gastrointestinal injuries. These bristles are often ingested unknowingly and can migrate to the liver, pancreas, or mesentery, sometimes causing abscesses or sepsis [12]. Some authors even advocate for banning these wire bristles to avoid these kinds of complications [12].

These cases exemplify the importance of multidisciplinary persistence in managing obscure abdominal pain with atypical imaging findings [13]. Delays in localization may require multiple modalities and re-evaluation. Furthermore, it highlights the need for increased public awareness about the dangers of wire-bristle ingestion [14].

## Conclusion

These two cases highlight the diagnostic and therapeutic challenges of gastric perforations caused by ingested foreign bodies, particularly difficult-to-detect metal bristles from grill brushes. Despite nonspecific clinical findings and inconclusive initial endoscopy in one case, persistent CT imaging and subsequent laparoscopic exploration ultimately enabled successful management.
